# Competition constrains parasite adaptation to thermal heterogeneity

**DOI:** 10.1093/evlett/qrag016

**Published:** 2026-04-22

**Authors:** Samuel TE Greenrod, Daniel Cazares, Weronika Ślesak, Tobias E Hector, R Craig MacLean, Kayla C King

**Affiliations:** Department of Biology, University of Oxford, Oxford, United Kingdom; Department of Biology, University of Oxford, Oxford, United Kingdom; Department of Biology, University of Oxford, Oxford, United Kingdom; Department of Biology, University of Oxford, Oxford, United Kingdom; Department of Biology, University of Oxford, Oxford, United Kingdom; All Souls College, High Street, Oxford, United Kingdom; Department of Biology, University of Oxford, Oxford, United Kingdom; Department of Zoology, University of British Columbia, Vancouver, Canada; Department of Microbiology & Immunology, University of British Columbia, Vancouver, Canada

**Keywords:** temperature, thermal heterogeneity, competition, evolution, co-selection, phage

## Abstract

Temporal thermal heterogeneity is expected to favour intermediate, generalist phenotypes that can maintain growth across a broad thermal range but have sub-optimal growth at any single temperature. Yet, thermal variation typically occurs in the presence of additional selection pressures which may interact to constrain adaptation to temperature. We propagated competing lytic viral parasites (bacteriophages, thermal specialist φ14–1 and thermal generalist φLUZ19) of *Pseudomonas aeruginosa* under fluctuating temperatures (37°C–42°C) in monoculture and in co-culture. Without competition, fluctuating temperatures resulted in intermediate thermal phenotypes in the phage φ14–1 and, in both phages, resulted in more variable evolutionary outcomes compared to static conditions. Selection from both fluctuating temperatures and competition accelerated thermal adaptation in the phage φ14–1. However, co-selection led to restricted thermal adaptation, lower genetic distance from ancestor, and fewer putative adaptive mutations in the phage φLUZ19. Our study highlights the potential for variable adaptive capacity in interacting communities amidst global climate change.

## Introduction

Thermal heterogeneity plays a key role in shaping species’ evolutionary trajectories. Spanning a broad range of timescales, temperatures fluctuate across multi-year periods (ENSO), between seasons, and even on the order of hours through diurnal (24-hour) cycles. Very slow or rapid thermal fluctuation frequencies, with respect to generation times, typically lead to similar adaptation to static environments through selective sweeps by specialist variants (Kassen, 2002; Sachdeva et al., 2020). Moderate fluctuation frequencies select for thermal generalists which have intermediate phenotypes across temperatures (Kassen, 2002; Kassen & Bell, 1998; Sachdeva et al., 2020). Generalist phenotypes often arise through the acquisition of multiple specialist mutations (Lambros et al., 2021) or single pleiotropic mutations (Sandberg et al., 2017). Thermal heterogeneity can also promote diversifying selection (Abdul-Rahman et al., 2021; Chesson, 2000; Harrison et al., 2013) leading to the maintenance of thermal specialist sub-populations (Harrison et al., 2013). The mechanisms of adaptation to thermal heterogeneity depend on the fluctuation frequency relative to generation time (Gilchrist, 1995). Fluctuations that exceed generation times in fast-reproducing species may favour specialists, but in slow-reproducing species may instead select for generalists.

Thermal heterogeneity typically occurs in the context of multiple selective pressures. For example, fluctuating temperatures can impose selection on species that are simultaneously adapting to other abiotic factors (e.g., pH (Krishna et al., 2025)) or to interactions with predators, competitors, or antagonists (Gunderson et al., 2016; Hector et al., 2022). The presence of multiple selection pressures can constrain evolution rates through combined negative effects on species fitness which reduce population sizes and mutational supply (Crain et al., 2008; Hiltunen et al., 2018). Co-selection can also restrict adaptation through pleiotropic fitness trade-offs; high fitness under one factor reduces fitness under another (Burmeister et al., 2020; Schou et al., 2022). Temporal thermal heterogeneity is expected to promote genetic diversification by increasing niche differences (Yamamichi et al., 2023) and so may offset the diversity-suppressing impacts of co-selection. However, some studies have indicated that co-selection involving temporal heterogeneity can exacerbate evolutionary constraint (Barbosa et al., 2014; Cairns et al., 2022; Uiterwaal et al., 2020). The ability of species to adapt to thermal heterogeneity amidst other selection pressures plays an important role in the maintenance of global biodiversity and species extinction risk (Bellard et al., 2012; Crain et al., 2008; Halpern et al., 2008; Vinebrooke et al., 2004).

Parasites provide an ideal group of organisms to study adaptation to thermal heterogeneity. Parasites are often exposed to diverse environments and stressors across multi-stage life cycles. They can have both free-living, vector-based, and host-associated life stages (Nguyen & Gokhale, 2025). By moving through numerous external environments during and between replicative cycles, parasites experience high temporal thermal heterogeneity (Greenrod et al., 2025b; Silva et al., 2025). During the infection stage, parasites can also induce fevers in hosts, driving thermal changes (Oakley et al., 2011). Finally, parasites are expected to face increasingly frequent thermal extremes as a result of global climate change (Carlson et al., 2017). While contending with variable thermal environments, parasites must adapt to host immune responses (Buckingham & Ashby, 2022) and competition with co-infecting parasites in the same host population or individual (Pedersen & Fenton, 2007). Within and between-host competition are primary determinants of parasite virulence (Hasik et al., 2023) signifying that interactions between competition and environment-based selection can shape parasite evolution (Limberger & Fussmann, 2021).

We predicted that thermal heterogeneity would select for generalist parasite populations with intermediate phenotypes and promote genetic diversity (Kassen, 2002). We also predicted that co-selection with other environmental stressors would constrain parasite adaptation (Cairns et al., 2022). We passaged two lytic viral parasites (thermal generalist φLUZ19 and specialist φ14–1) under a fluctuating thermal regime (37–42°C) in the absence and presence of a phage competitor. Phages evolved with a static bacterial host, *Pseudomonas aeruginosa*. We compared populations evolved under fluctuating temperatures concurrently with those evolved under a static regime (37°C and 42°C), the latter presented in ref. (Greenrod et al., 2025a). We evaluated phage phenotypic adaptation through growth assays at 37°C and 42°C. We also conducted phage population sequencing to identify adaptive mutations and measure genetic distance from the ancestor.

## Methods and materials

### Strains, storage, and culture conditions

This study builds on a previously published experimental framework (Greenrod et al., 2025a) using the same bacterial host and bacteriophage strains. *Pseudomonas aeruginosa* PAO1 was used as the non-evolving bacterial host throughout. Two lytic phages, φLUZ19 and φ14–1, were used due to their known thermal response differences: φLUZ19 performs well at both 37°C and 42°C, while φ14–1 is growth-restricted at 42°C (Greenrod et al., 2024, 2025a). Phage lysates and bacterial stocks were prepared as in refs (Greenrod et al., 2024, 2025a).

### Experimental evolution

The experimental evolution design closely followed that of ref. (Greenrod et al., 2025a) with additional treatments incorporating fluctuating temperatures. Phages were serially passaged for 15 days under four conditions: monoculture and co-culture, each at either static or fluctuating temperatures (daily shifts between 37°C and 42°C). Each treatment included six independent replicate populations, and all populations were initiated from a single ancestral lysate.

Phages were propagated without shaking with a non-evolving ancestral PAO1 bacterial host. For the initial passage, ancestral phage lysates were diluted to 10^8^ PFU/ml and 300μl were added to 2.7ml 10^8^ CFU/ml bacterial culture in loose-lid 14ml falcon tubes. Phage co-culture populations were prepared by combining 150μl each of φLUZ19 and φ14–1 10^8^ PFU/ml lysates prior to mixing with bacteria. The initial passage phage densities were ∼10^7^ PFU/ml resulting in a phage/bacteria ratio (multiplicity of infection, MOI) = ∼0.1. Following addition of bacterial cultures, tubes were incubated statically at 37°C or 42°C in circulating water baths for 8h. Fluctuating passages started and ended at 37°C.

After incubation, phage populations were harvested by centrifugation (3,095 × g, 5 min) to pellet bacteria, followed by sterile filtration of the supernatant through 0.2μm filters. Filtrates were then stored at 4°C overnight. In subsequent passages, 300μl of lysate was transferred into fresh PAO1 cultures. One 8h passage was performed per day leading to 15 passages in total.

### Phage quantification

Phage titres were determined via the double-layer overlay method (Kropinski et al., 2009) following the same protocols as in refs (Greenrod et al., 2024, 2025a). Briefly, bacterial lawns were prepared by mixing 10mL of melted LB-top agar with 300µL of a *P. aeruginosa* PAO1 overnight culture. Phage lysates were serially diluted, and 10µL was spotted onto the bacterial lawns. After incubating plates for 6–8 h at 37°C, spots with the highest number of discernible plaques were counted and reported. φLUZ19- or φ14–1-resistant PAO1 strains were used for selective plating enabling separate counting of φLUZ19 and φ14–1 densities in co-cultures. These resistant strains were derived by isolating colonies growing on high titre phage plaques and confirmed via sequencing (Greenrod et al., 2025a). All monoculture and co-culture samples were quantified using the appropriate resistant strains to ensure consistency.

### Phage separation and concentration

To generate high-titre and pure phage lysates for downstream assays and sequencing, we employed selective double-layer overlays with resistant hosts. Briefly, phages and φLUZ19- or φ14–1-resistant PAO1 strains (from (Greenrod et al., 2025a)) were seeded into top agar plates to allow phage propagation. Phages were extracted from plates by scraping top-agar into 15ml falcon tubes containing 5ml of phage buffer (NaCl (100 mM), MgSO_4_ (10 mM), CaCl_2_ (5 mM), Tris-HCl (pH 8) (50 mM), Gelatin (0.01%)). Tubes were mixed overnight after which phages were separated from top agar using sterile-filtration. This process was performed three times to ensure removal of phage competitors from co-culture populations. The purification and extraction protocols were identical to those described in ref. (Greenrod et al., 2025a).

### Phage growth rate assays

The thermal phenotypes of purified evolved phage populations relative to the ancestor were assessed by measuring phage densities across an 8h window under static incubation at 37°C and 42°C. Phage lysates were diluted to 10^5^ PFU/ml and 300uL was mixed with 2.7ml of 10^8^ CFU/ml wild-type PAO1 to a final MOI = ∼0.0001. φLUZ19 was sampled at 2h, 4h, and 8h; φ14–1 at 4h and 8h due to delayed replication. Phage quantification was performed through sterile-filtration through 0.22μm filter plates (Agilent) followed by centrifugation at 2,230xg for 5 mins before spotting onto resistant PAO1 double-layer overlay plates. Phage growth rate was determined based on phage densities after 2h of growth (φLUZ19) and 4h of growth (φ14–1). Given phage populations started at equal densities, populations with higher densities after growth are regarded as having faster growth rates. Each growth rate assay included a single replicate of each evolved phage population and three replicates of the phage ancestor. Growth rate assays were repeated three times across a two-week period to produce three technical replicates.

### Phage population genomics

#### DNA extraction and sequencing

Phage DNA was extracted from purified lysates as described in (Greenrod et al., 2025a). Briefly, ancestral and evolved phage lysates were treated with DNase and RNase to remove bacterial DNA and RNA. Phage particles were lysed using lysis (AL) buffer and proteinase K. Cell debris was precipitated using precipitation (N4) buffer and removed. Finally, DNA was precipitated and washed using isopropanol and ethanol. DNA quality was assessed with NanoDrop 2000c (Thermo Scientific) and quantified with Qubit 4 (Thermofisher). Paired-end 2 × 250bp read Illumina sequencing was performed by AZENTA/GENEWIZ using their Microbe-EZ pipeline for evolved and ancestral populations. One extraction was performed for each evolved population and three extractions were performed for each phage ancestor. Bacterial genomes (wild-type and phage-resistant strains) were sequenced by MicrobesNG using hybrid (long- and short-read) approaches.

#### Sequence analysis

Phage reads were pre-processed with Trim Galore (v.0.5.0) (https://github.com/FelixKrueger/TrimGalore) using default settings. As read depth was uneven across the phage genome, reads were downsampled to a target read depth of 1500x and a minimum depth of 1000x using bbnorm from the bbmap package (v.39.18) (https://sourceforge.net/projects/bbmap/). Ancestral phage genomes were assembled using shovill (v1.1.0) (https://github.com/tseemann/shovill) with default parameters. Ancestral assemblies were annotated with prokka (v.1.14.5) (Seemann, 2014), guided by the NCBI GenBank file for each phage (φ14–1: NC_011703; φLUZ19: NC_010326). Phage reads were mapped to de novo ancestral assemblies using Bowtie2 (v.2.3.4.2) (Langmead & Salzberg, 2012). Variants were identified using breseq (v.0.36.1) (Deatherage & Barrick, 2014) in polymorphism mode, using the annotated ancestral genomes as a reference. A standard polymorphism E-value was used as a threshold for variant calling (E ≤ 10^−2^). Only variants with > 10% allele frequency (∼100–150 read support) were analysed. Potential sequencing biases were accounted for by removing variants from evolved populations that co-occurred in the ancestral population. Genetic distance between evolved populations and from the ancestor were determined by calculating Euclidean distances between populations based on the frequency of variants at > 10% allele frequency.

Wild-type and resistant PAO1 genomes were assembled using Autocycler (v. 0.4.0) (Wick et al., 2025) and polished via Polypolish (v. 0.6.0) (Wick & Holt, 2022). Final assemblies were re-oriented with Dnaapler (v. 1.2.0) (Bouras et al., 2024) and annotated using prokka (v.1.14.5) (Seemann, 2014). The workflow was deployed using a Dockerised Nextflow pipeline (v. 1.0.2) available at https://doi.org/10.5281/zenodo.15706447. Mutations in resistant PAO1 strains were identified by mapping long reads to the wild-type assembly with minimap2 (v.2.24) (Li, 2018) and variant calling with medaka (v.2.1) (https://github.com/nanoporetech/medaka). All bioinformatic analyses were conducted with default parameters.

### Statistical analyses and visualisation

All statistical analyses and data visualisation were conducted using packages in R (v.4.3.2) and RStudio (R Core Team, 2025; Posit Team, 2025). Data wrangling was performed using “Tidyverse” (v.2.0.0) R packages (Wickham et al., 2019). Phage per-population total progeny, growth rates, and genetic distance from ancestor were compared between evolution treatments using linear mixed effect models with the “lme4” (v.1.1–36) R package (Bates et al., 2015) where the response variable was total phage progeny, phage density (pfu/ml) after 2h (φLUZ19) or 4h (φ14–1) of growth, or genetic distance from ancestor, respectively. Estimates are provided in log_10_ units as phage densities were logged prior to analysis to meet statistical assumptions. Explanatory variables were an interaction term between evolution treatment and temperature, with batch (technical replicate) as a random effect. Genetic distance from ancestor was determined by using the “dist” function in the “stats” (v.4.5.2) R package. Pairwise comparisons between populations were determined for each temperature separately using the “emmeans” (v.2.0.0) (Lenth & Piaskowski, 2025) R package. Separate models were run for each phage. Within-group variation in genetic distance from ancestor was analysed using Levene’s test. The prevalence of unique compared to shared mutations across evolution treatments was analysed using Fisher’s exact test. Phage genetic distance between groups was also compared by constructing neighbour-joining trees based on Euclidean genetic distance using the “ggtree” (v.3.10.1) R package (Yu et al., 2017).

## Results

### Fluctuating temperatures select for generalist phenotypes in monoculture

**Figure 1:**
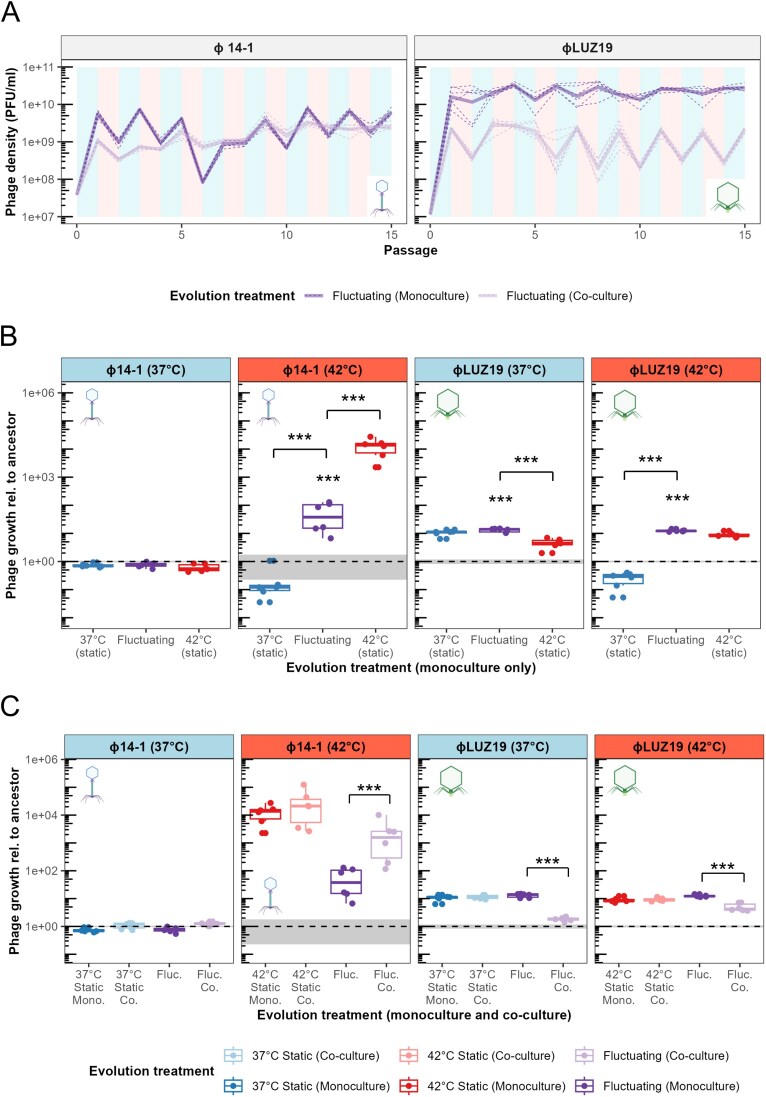
Co-selection in communities constrains adaptation to thermal fluctuations. (**A**) Population dynamics of phages passaged in monoculture and co-culture under fluctuating temperatures. Values show densities at the end of each passage prior to dilution. As phage lysates were diluted 10-fold in between passages, density decreases reflect less than 10-fold population growth during passages. Plot background colour shows alternating 37-42°C passage temperatures, starting and ending at 37°C. Phage icons illustrate the two different phages used in the experiments (φ14–1, myovirus as tall phage; φLUZ19, autographivirus as short phage) (Tabare et al., 2021) and are used hereafter to refer to phages in figures. (**B**) Fluctuating and static temperature-evolved population growth rates relative (rel.) to the ancestor. Growth rates were measured after 2h for φLUZ19 and 4h for φ14–1. While plot shows growth relative to ancestor, statistical analysis directly compared evolved and ancestral population growth rates. Six biological replicates were assayed, and data points show the average of three technical replicates (batches). Technical replicates were not averaged in the statistical model but were included as a random effect. Growth assay temperature is shown in panel strips and represented by panel strip colour. Ancestral growth is shown by dashed line with standard errors shown as a shaded box (n = 3). *** = *p* < 0.001. Asterisks above boxes show statistical comparisons with the ancestor. Asterisks above contrast lines show statistical comparisons between evolved populations. Absence of asterisk reflects non-significance. Static monoculture temperature data was adapted from ref. (Greenrod et al., 2025a). (**C**) Growth rates of fluctuating and static temperature monoculture-evolved populations compared to co-culture evolved populations. Boxes and points are coloured by evolution treatment with monoculture labels abbreviated to Mono. and co-culture labelos abbreviated to Co. Labels for fluctuating temperature-evolved populations are abbreviated to Fluc. Assay temperature and significance values are presented as in Figure 1B. Six biological replicates were assayed and data points show the average of three technical replicates (batches). Technical replicates were not averaged in the statistical model but were included as a random effect. Ancestral growth rates and significance signs are presented as in Figure 1B. Static co-culture data was adapted from ref. (Greenrod et al., 2025a).

Fluctuating environments can favour generalists with intermediate phenotypes across conditions (Kassen, 2002). Given the ancestral φ14–1 has previously been shown to grow very poorly at 42°C (Greenrod et al., 2025a), we hypothesised that φ14–1 populations passaged under fluctuating conditions would rapidly adapt to 42°C but have lower fitness at 37°C and 42°C compared to static evolved populations. In monoculture, φ14–1 densities increased during 37°C passages but decreased in 42°C passages (Figure 1A). As phage lysates were diluted 10-fold in between passages, phage density decreases reflect lower than 10-fold φ14–1 population growth during 42°C passages. φLUZ19 monoculture populations reached and then maintained high densities in all passages. This phage showed low variation in inter-passage densities. We assessed evolutionary potential by measuring the total number of progeny produced by each phage population across the experiment (Fig. S1). We did not observe a significant difference in per-population total progeny between evolution treatments in monoculture for either phage.

We assessed phage evolution by measuring the growth rates of static and fluctuating evolved populations at 37°C and 42°C. Growth rates were determined by measuring population densities after 2h (φLUZ19) and 4h (φ14–1) of growth (see methods for details). Results are presented in Figure 1B & C as relative to the ancestral phage although evolved and ancestral population densities were directly compared in statistical analyses. We found that growth rates of both phages in monoculture depended on the interaction between evolution treatment and assay temperature (φ14–1: F_3,116_ = 238.6, *p* < 0.001; φLUZ19: F_3,116_ = 137.4, *p* < 0.001) (Figure 1B). At 42°C, φ14–1 fluctuating populations were found to have an intermediate phenotype between those evolved under static conditions. φ14–1 fluctuating populations had significantly higher growth rates at 42°C than 37°C static populations (37°C static vs fluctuating: estimate = -2.6 (log_10_), t(116) = –17.6, *p* < 0.001) but lower growth rates than 42°C static populations (fluctuating vs 42°C static: estimate = 2.5 (log_10_), t(116) = 16.8, *p* < 0.001). At 37°C, φ14–1 fluctuating populations had no significant difference to static populations possibly due to phage growth being measured after phages had reached carrying capacity (see ref. (Greenrod et al., 2025a)). For φLUZ19, fluctuating evolved populations had significantly higher growth at 42°C than 37°C static populations (37°C static vs fluctuating: estimate = -1.9 (log_10_), t(116) = -22.8, *p* < 0.001). However, growth was not significantly different to 42°C evolved populations (fluctuating vs 42°C static: estimate = -0.15 (log_10_), t(116) =-1.79, *p* = 0.28). The opposite findings were observed at 37°C; fluctuating evolved populations had significantly higher growth rates than those evolved at 42°C but similar growth rates to 37°C evolved populations (42°C static vs fluctuating:: estimate = -0.52 (log_10_), t(116) = -6.1, *p* < 0.001; 37°C static vs fluctuating: estimate = -0.07 (log_10_), t(29) = -0.84, *p* = 0.83).

### Co-selection from fluctuating temperatures and competition constrains thermal adaptation

The presence of additional selection pressures is expected to constrain adaptation to fluctuating temperatures by reducing mutational supply and compounding fitness trade-offs (Barbosa et al., 2014; Crain et al., 2008; Uiterwaal et al., 2020). Indeed, phage populations evolved in co-culture had significantly lower per-population total progeny than those evolved in monoculture for both φ14–1 and φLUZ19 (φ14–1: 37°C static co. vs mono: estimate = -0.93 (log_10_), t(30) = -9.9, *p* < 0.001; 42°C static co. vs mono: estimate = -0.33 (log_10_), t(30) = -3.6, *p* < 0.05; fluctuating co. vs mono: estimate = -0.65, t(30) = -7.0, *p* < 0.0001; φLUZ19: 37°C static co. vs mono: estimate = -2.0 (log_10_), t(30) = -25.6, *p* < 0.001; 42°C static co. vs mono: estimate = -2.6 (log_10_), t(30) = -32.2, *p* < 0.001; fluctuating co. vs mono: estimate = -2.7, t(30) = -33.8, *p* < 0.0001) (Fig. S1). We hypothesised that phages evolved under co-selection from fluctuating temperatures and competition would have lower growth rates at 37°C and 42°C than those evolved under static temperatures or fluctuating monoculture conditions. While φ14–1 densities fluctuated between passages in monoculture, co-culture densities rapidly increased and then stabilised between passages (Figure 1A). In contrast, φLUZ19 populations were stable in monoculture, but during fluctuations in co-culture, experienced high growth at 37°C and low growth at 42°C.

We then assessed co-culture and monoculture evolved phage growth rates at 37°C and 42°C (Figure 1C). We found a significant interaction between evolution treatment (monoculture and co-culture) and temperature regarding growth rates for both phages (φ14–1: F_6,218_ = 178.9, *p* < 0.0001; φLUZ19: F_6,218_ = 123.2, *p* < 0.0001). While competition had no impact on the growth rates of static φ14–1 populations at their respective evolved temperatures (37°C static mono vs comp: estimate = -0.41 (log_10_), t(218) = -1.2, *p* = 0.89; 42°C static mono vs comp: estimate = -0.44 (log_10_), t(218) = -1.3, *p* = 0.85), fluctuating co-culture evolved populations had significantly higher growth rates at 42°C compared to populations evolved in monoculture (fluctuating mono vs comp: estimate = -3.4 (log_10_), t(218) = -10.1, *p* < 0.0001). No significant difference was observed at 37°C (fluctuating mono vs comp: estimate = -0.54 (log_10_), t(218) = -1.6, *p* = 0.69). Similar to φ14–1, there was no impact of competition on static evolved φLUZ19 population growth rates at their respective evolved temperatures (37°C static mono vs comp: estimate = 0.07 (log_10_), t(218) = 0.87, *p* = 0.97; 42°C static mono vs comp: estimate = -0.02 (log_10_), t(218) = -0.20, *p* = 1.0). However, φLUZ19 populations evolved with fluctuations and competition had significantly lower growth rates at both 37°C and 42°C compared to monoculture (fluctuating mono vs comp 37°C: estimate = 0.82 (log_10_), t(218) = 10.2, *p* < 0. 0001; fluctuating mono vs comp 42°C: estimate = 0.48 (log_10_), t(218) = 6.1, *p* < 0.001).

### Fluctuating environments favour specialist mutations

**Figure 2:**
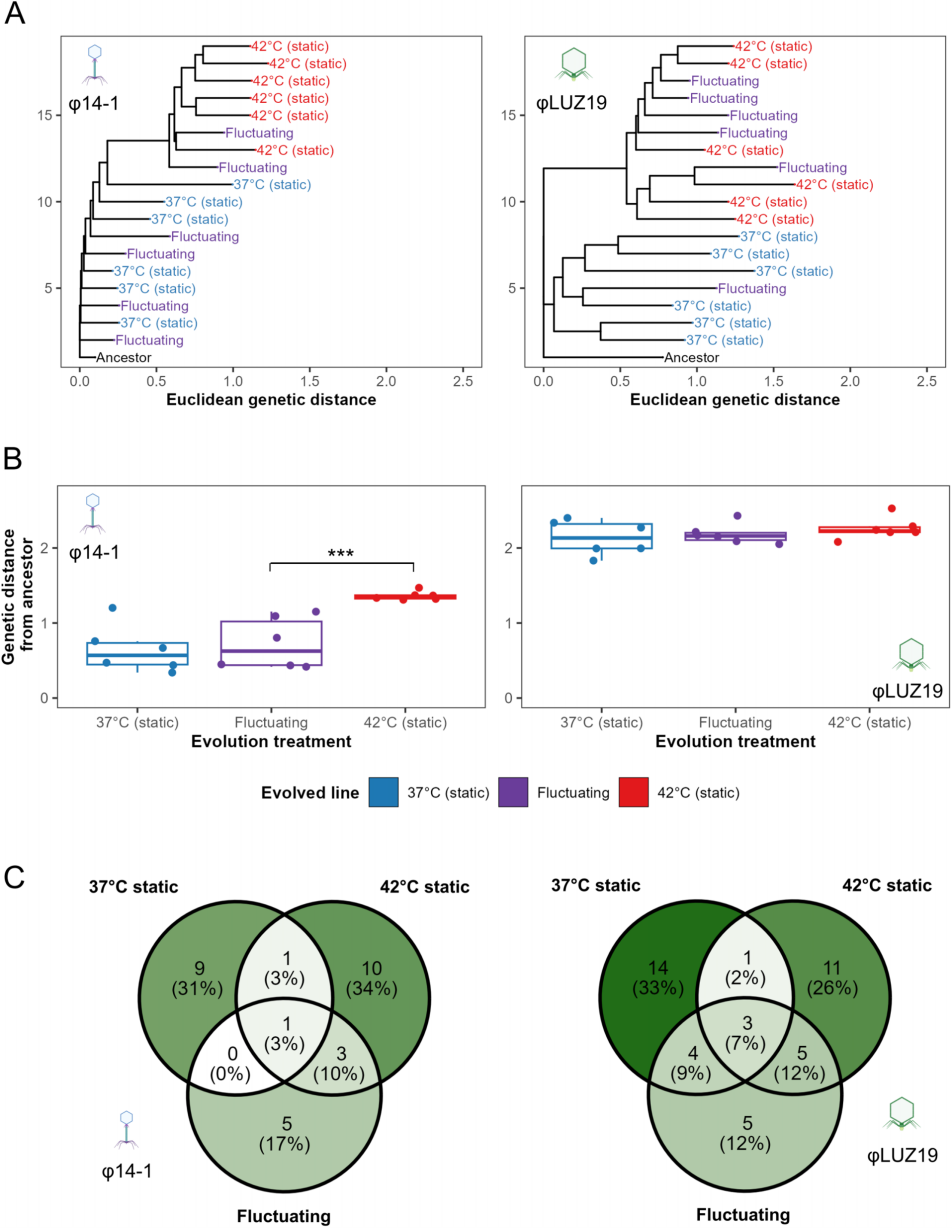
Fluctuating temperatures select for specialist mutations. (**A**) Neighbour-joining trees of evolved and ancestral phage populations constructed using Euclidean genetic distances. Genetic distances were calculated based on the presence and frequency of mutations present at > 10% frequency. Tree is rooted at the ancestor and populations are coloured by evolved treatment. (**B**) Phage Euclidean genetic distance from the ancestor for evolved monoculture phage populations. *** = *p* < 0.001. (**C**) Venn diagrams show the number of high frequency (>20% frequency), putative adaptive variants that are unique to or shared between evolution treatments. Venn diagram sections are coloured based on the number of variants. Static temperature data was adapted from ref. (Greenrod et al., 2025a).

Fluctuating temperatures generally select for multiple specialist mutations (Lambros et al., 2021). We hypothesised that fluctuating evolved populations would show genetic similarities to both high and low temperature static populations. Phage genomic evolution was assessed by constructing neighbour-joining trees of end-point populations based on Euclidean genetic distances (Figure 2A). Genetic distances were calculated based on the presence and frequency of genetic variants (SNPs, indels) that had > 10% frequency. Fluctuating evolved populations did not form a unique clade but instead were found to co-locate with either high or low temperature static populations. φ14–1 fluctuating populations were distributed across the tree and generally did not cluster with static populations. Conversely, φLUZ19 fluctuating populations were primarily found within the 42°C static clade.

We further analysed static and fluctuating population genetic similarities by measuring Euclidean genetic distance from ancestor (Figure 2B). For φ14–1, fluctuating evolved populations had significantly lower genetic distances from ancestor than 42°C static populations (t(15) = -4.1, *p* < 0.01). However, there were no significant differences in genetic distance from ancestor between fluctuating and 37°C static populations (t(15) = -0.51, *p* = 0.87). There were no significant differences in genetic distance from ancestor between φLUZ19 fluctuating populations and either 37°C or 42°C static populations (37°C: t(15) = -0.46, *p* = 0.89; 42°C: t(15) = -0.75, *p* = 0.74). φ14–1 fluctuating populations had significantly greater within-group variation in genetic distance from ancestor compared to 42°C static populations (t(15) = 2.9, *p* < 0.05) but not 37°C static populations (t(15) = -0.70, *p* = 0.77). φLUZ19 fluctuating populations had no significant difference in within-group variation compared to static populations (37°C: t(15) = 2.0, *p* = 0.15; 42°C: t(15) = -0.10, *p* = 0.99). The number of variants in each phage population can be found in Figure S2.

We then determined the prevalence of individual genetic variants (SNPs, indels) that were unique to or shared between evolution treatments (Figure 2C). Only putative adaptive variants with > 20% frequency were included. For φ14–1, 2/11 (18%) of 37°C static variants and 5/15 (33%) of 42°C static variants were shared with other evolution treatments compared to 4/9 (44%) variants in fluctuating evolved populations. For φLUZ19, shared variants constituted 8/32 (35%) of 37°C static and 9/20 (45%) of 42°C static variants compared to 12/17 (71%) in fluctuating evolved populations. To assess the overall impact of evolution treatment on the ratio of unique and shared mutations, we pooled mutations from φ14–1 and φLUZ19 observing a significant difference in the prevalence of shared mutations relative to unique mutations between evolution treatments (Fisher’s exact test: *p* < 0.05). Significant differences were not observed when analysing phages independently (φ14–1, *p* = 0.47; φLUZ19, *p* = 0.08). φ14–1 fluctuating mutations were primarily shared with 42°C static populations. In contrast, φLUZ19 fluctuating mutations were shared equally with 37°C and 42°C static populations.

Finally, we investigated which mutations drove clustering between fluctuating and static populations (Fig. S3; Table S1). While φ14–1 fluctuating and 37°C static populations showed little clustering, two fluctuating populations clustered with the 42°C static clade. These two replicate populations contained parallel deletions in a hypothetical protein with high similarity to a DNA ligase (BlastP: 97.47% identity, 95% sequence overlap with Pseudomonas phage PhL_UNISO_PA-DSM_ph0031 DNA ligase protein), previously identified in all φ14–1 42°C static populations (Greenrod et al., 2025a). As found with static evolved populations (Greenrod et al., 2025a), all fluctuating φLUZ19 populations acquired mutations in tail fiber genes indicating selection on these genes is strong but independent of thermal regime. The clustering of 5/6 φLUZ19 fluctuating populations with the 42°C static populations were attributed to parallel insertions in an intergenic region between two hypothetical proteins. This intergenic insertion was previously identified in all φLUZ19 42°C static populations (Greenrod et al., 2025a).

### Co-selection constrains molecular evolution

**Figure 3:**
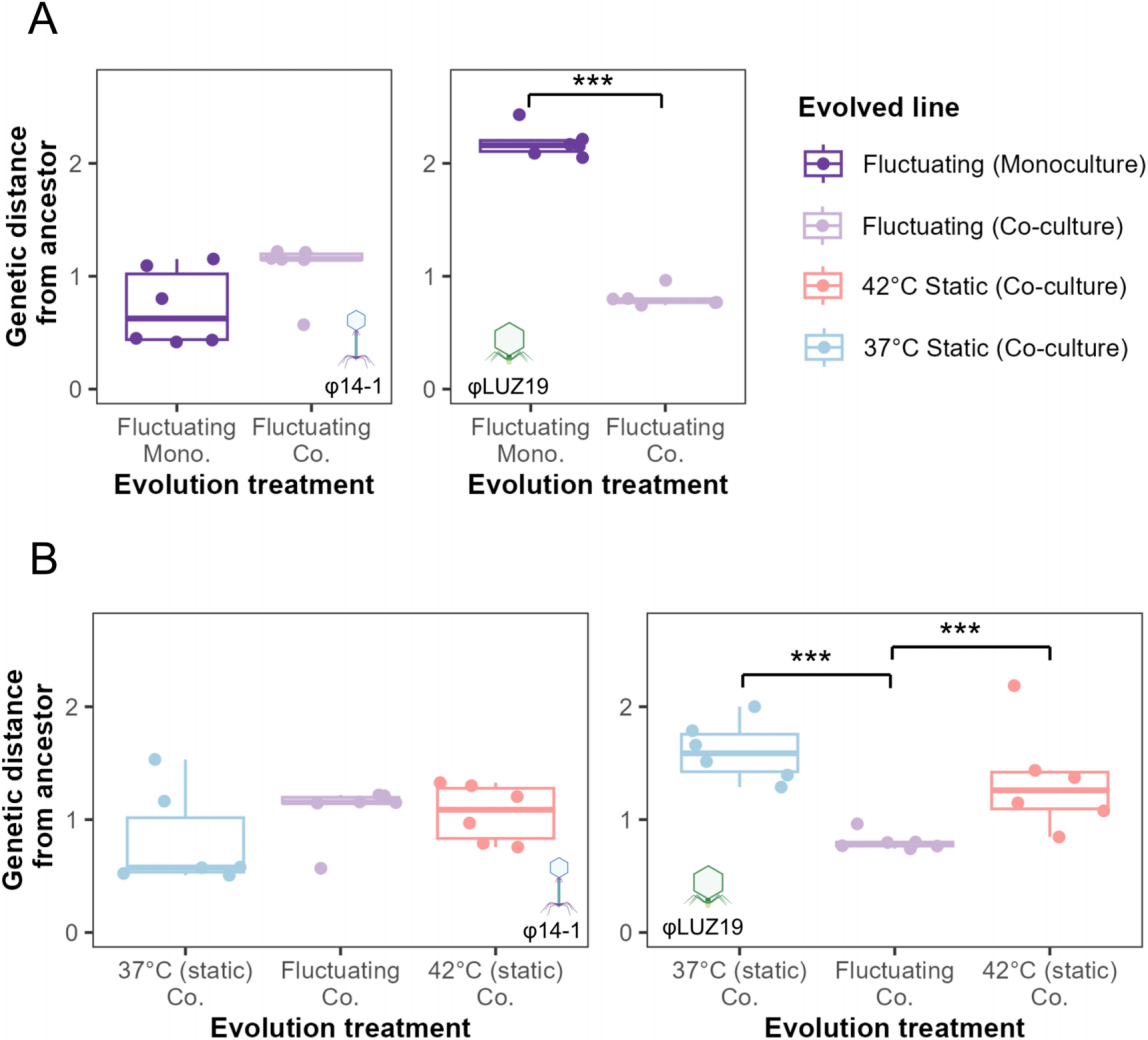
High environmental complexity constrains genetic distance from ancestor in the φLUZ19 phage. (**A**) Euclidean genetic distance from the ancestor for phage populations evolved under fluctuating temperatures in monoculture (labelled Mono.) and co-culture (labelled Co.). (**B**) Genetic distance from ancestor for 37°C static, fluctuating, and 42°C static co-culture populations. Plot layout is the same as panel A. *** = *p* < 0.001. Static temperature data was adapted from ref. (Greenrod et al., 2025a).

By reducing population growth rates, co-selection from fluctuating temperatures and competition are expected to reduce genetic distance from ancestor and restrict the acquisition of adaptive mutations (Crain et al., 2008). Due to their elevated growth rates, we hypothesised that φ14–1 fluctuating co-culture populations would have higher genetic distances from ancestor than monoculture populations. No significant differences in genetic distance from ancestor were observed for φ14–1 co-culture populations compared to monoculture populations (F_1,10_ = 4.1, *p* = 0.07) (Figure 3A). However, non-significance was driven by a single low genetic distance replicate in the co-culture treatment; when the replicate was removed, co-culture populations had significantly greater genetic distances from ancestor than monoculture populations (F_1,9_ = 8.6, *p* < 0.05). We also found no significant difference in within-group variation in genetic distance from ancestor between monoculture and co-culture populations (t(10) = 1.50, *p* = 0.17), although the difference was also significant once the low genetic distance co-culture replicate was removed (t(10) = 3.6, *p* < 0.01).

For φLUZ19, we hypothesised that fluctuating co-culture populations would have lower genetic distances from ancestor than monoculture populations due to the suppression of φLUZ19 by φ14–1 (Fig. S1). φLUZ19 fluctuating co-culture populations had significantly lower genetic distances from ancestor than monoculture populations (F_1,10_ = 470, *p* < 0.001). We further hypothesised that φLUZ19 fluctuating co-culture populations, but not φ14–1 populations, would have lower genetic distances from ancestor than 37°C or 42°C static co-culture populations. Genetic distances from ancestor were equal between co-culture populations for φ14–1 (F_2,15_ = 1.2, *p* = 0.3) (Figure 3B). However, φLUZ19 fluctuating co-culture populations were found to have significantly lower genetic distances from ancestor than both 37°C static and 42°C static monoculture populations (37°C: t(15) = 4.5, *p* < 0.01; 42°C: t(30) = 3.0, *p* < 0.05).

Finally, we assessed the impact of competition on the acquisition of high frequency mutations (> 20% frequency) in fluctuating populations (Fig. S4). For φ14–1, while populations no longer acquired singleton mutations, all populations acquired a deletion or SNP in a putative DNA ligase gene. Mutations in this gene are thought to contribute to high temperature adaptation (Greenrod et al., 2025a) and were also found in the two monoculture populations which clustered with 42°C static populations in Figure 2A. For φLUZ19, while co-culture populations maintained mutations in tail fiber genes, the populations no longer acquired singleton mutations or the intergenic insertion associated with 42°C static populations.

## Discussion

Under fluctuating selection, both phages were found to rapidly evolve increased growth rates at high temperatures. For phage φ14–1, fluctuating temperatures resulted in intermediate thermal phenotypes with lower growth rates at high temperatures compared to phages evolved under static temperatures. The evolution of intermediate growth rates is likely due to weaker selection for adaptation to high temperatures in the fluctuating treatment. Alternatively, adaptation to high temperatures may be constrained during fluctuations due to fitness trade-offs at lower temperatures (Visher & Boots, 2020). For phage φLUZ19, fluctuating temperature populations had the same growth rates as static-evolved populations when measured at their evolved temperature. Given φLUZ19 was shown to exhibit growth rate trade-offs under static selection (Greenrod et al., 2025a), this finding is indicative of a no-cost generalist strategy (Remold, 2012). The lack of growth rate costs may reflect an epistatic pleiotropy, whereby the costs of adaptive mutations depend on the genetic background (Remold, 2012). Costs may also occur in unmeasured traits such as virulence (Ashrafi et al., 2018) or tolerance to other stresses (Schou et al., 2022). These findings highlight that, while fluctuating temperatures select for greater high temperature growth, the exact phenotypic outcomes of fluctuating selection vary between taxa (Wolinska & King, 2009).

We found that fluctuating temperatures resulted in more variable evolutionary trajectories; while static evolved populations generally formed clusters, fluctuating populations were genetically similar to both 37°C and 42°C static evolved populations. Further, we found that parallel mutations acquired under fluctuating selection were the same as those previously identified in static evolved populations (Greenrod et al., 2025a). These findings could be explained by historical contingency (Blount et al., 2018) whereby a small number of random mutations that are adaptive at low or high temperatures become fixed in a subset of populations (Harrison et al., 2013). As phage populations only acquired a small number of mutations during the experiment, the early acquisition and fixation of large fitness effect mutations may have caused fluctuating populations to resemble those that evolved under static environments. Fluctuating environments have also been shown to select for mutations conferring fitness in the most extreme environment (Arribas et al., 2014). The clustering of φLUZ19 fluctuating populations with 42°C static populations likely reflects selective sweeps combined with asymmetrical selection where 42°C adaptive variants are fixed more rapidly. Diversification and allele fixation is an expected outcome of our experimental set-up where fluctuations occurred at a much longer frequency than phage generation times. If fluctuations had occurred at the same rate or faster than generation times, we would expect less diversification through the evolution of plasticity or an increased risk of extinction (Botero et al., 2015; Kassen, 2002).

While fluctuating environments can promote genetic diversification, co-selection with other environmental stressors can constrain adaptation (Crain et al., 2008). Uiterwaal et al. (Uiterwaal et al., 2020) showed that combined fluctuating temperatures and predation restricted adaptation to both selection pressures in *Paramecium caudatum* populations. Similar findings have also been observed in *Daphnia magna* with co-selection from thermal fluctuations and predation/pollutants (Barbosa et al., 2014, 2017). We found that combined fluctuating temperature- and competition-based selection constrained both thermal adaptation and reduced genetic distance from ancestor in phage φLUZ19 but accelerated adaptation in φ14–1. Further, co-selection resulted in greater φLUZ19 evolutionary constraint with fluctuating temperatures than static temperatures. In a previous study, we demonstrated that competition accelerates thermal adaptation in φ14–1 but constrains genetic distance from ancestor in φLUZ19 (Greenrod et al., 2025a), indicative of a selective synergy (Crain et al., 2008). The present study extends these findings by showing that the selective synergy between temperature and competition is greater under fluctuating temperatures than static temperatures. One potential explanation for these findings is that fluctuating environments reduce the strength of selection for adaptive mutations (Cvijović et al., 2015). Weaker directional selection combined with suppression by competitors may constrain both the supply and fixation of beneficial mutations leading to particularly low genetic distances.

Anthropogenic activities, including global climate change, mean that species are facing increasingly variable and complex environments (Jaureguiberry et al., 2022). With ongoing global biodiversity loss, species must adapt to tolerate environmental stressors to avoid extinctions. Our findings highlight that while large-population species, such as viruses and bacteria, can rapidly adapt in response to thermal variation, co-selection with other factors, such as competition, may restrict species adaptive capacity. These results have particular relevance for parasites which, in addition to having large population sizes, must simultaneously adapt to both thermal heterogeneity, community competition in coinfections, and host immune responses (Altman et al., 2016; Mideo, 2009). With global parasite biodiversity at risk due to climate change (Carlson et al., 2017), evolutionary constraint caused by co-selection may prevent parasite adaptation to thermal stress and further increase the probability of parasite extinctions. The risk of extinction is likely higher for small population, slower growing species due to their slower evolution rates and the higher probability that fluctuations will occur within generations. Future studies should consider the evolution-constraining effects of co-selection when assessing species extinction risk in thermally variable environments.

## Supplementary Material

qrag016_Supplemental_Files

## Data Availability

Data and code used in analyses can be found at https://github.com/SamuelGreenrod/Evol_fluctuating. Fluctuating temperature-evolved phage sequence reads are accessible on NCBI (https://www.ncbi.nlm.nih.gov/) under BioProject ID: PRJNA1334331. Static temperature-evolved phage sequence reads and bacterial sequence reads from ref. (Greenrod et al., 2025a) are accessible under BioProject IDs: PRJNA1332698 and PRJNA1332799, respectively.
