# Supplementary material for: Competition constrains parasite adaptation to thermal heterogeneity
Source: Evol Lett. 2026 Apr 22;10(4):371–81. doi: 10.1093/evlett/qrag016 (PMC13451771; doi:10.1093/evlett/qrag016)
Supplement: Supplementary file 1 [file qrag016_supplemental_files.zip › Supplementary_figures.pdf]

Competition constrains parasite adaptation to thermal heterogeneity

Samuel TE Greenrod[1#], Daniel Cazares[1], Weronika Slesak[1], Tobias E Hector[1], R. Craig MacLean[1,2], Kayla C King[1,3,4,#]

[1] Department of Biology, University of Oxford, Oxford, UK

[2] All Souls College, High Street, Oxford OX1 4AL, UK

[3] Department of Zoology, University of British Columbia, Vancouver, Canada

[4] Department of Microbiology & Immunology, University of British Columbia, Vancouver, Canada

Supplementary figures

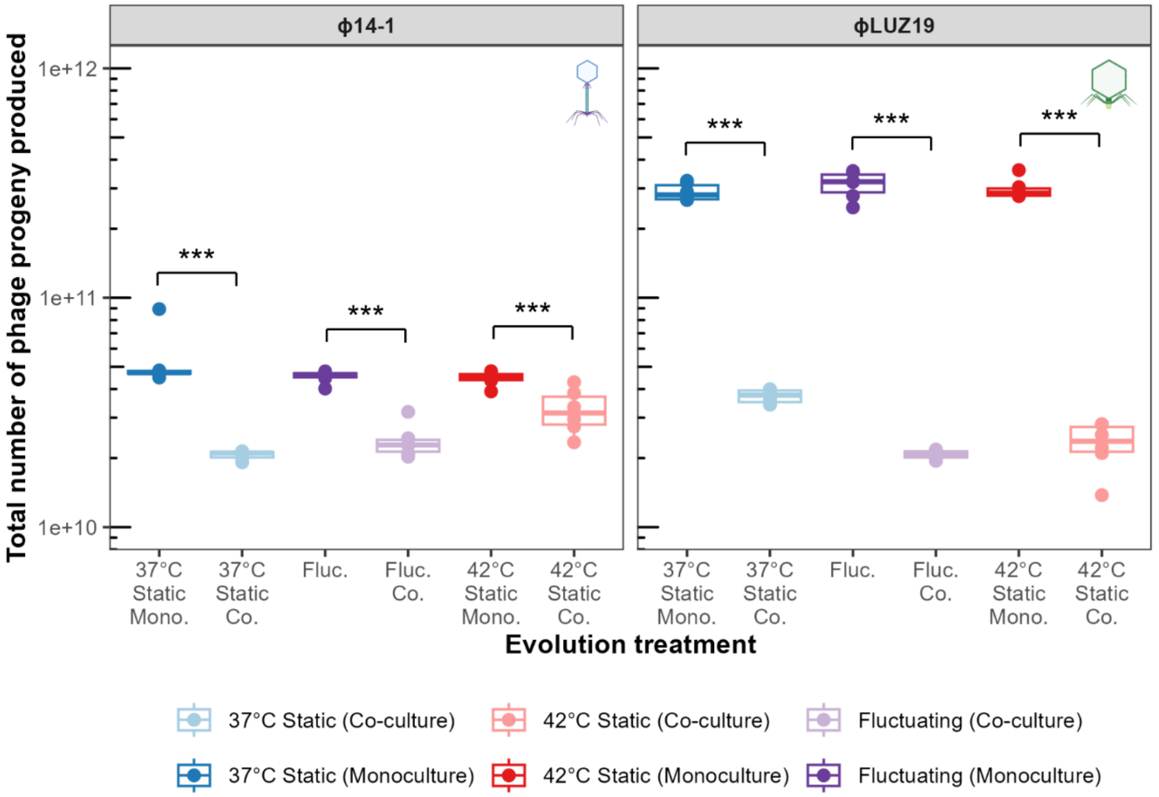

Figure S1. Per-population total phage progeny across experiment. Boxes and points show the sum total of phage progeny produced at each passage during the experiment based on data from Fig. 1A. Boxes and points are coloured by evolution treatment with monoculture in dark (37°C static in blue, 42°C static in red, and fluctuating in purple) and co-culture in light (37°C static in light blue, 42°C static in light red, and fluctuating in light purple). *** = $p < 0.001$. Lack of significance bar indicates non-significant differences. Six biological replicates were assayed and data points show the average of three technical replicates (batches). Technical replicates were not averaged in the statistical model but were included as a random effect. Static co-culture data was adapted from ref. (Greenrod et al., 2025).

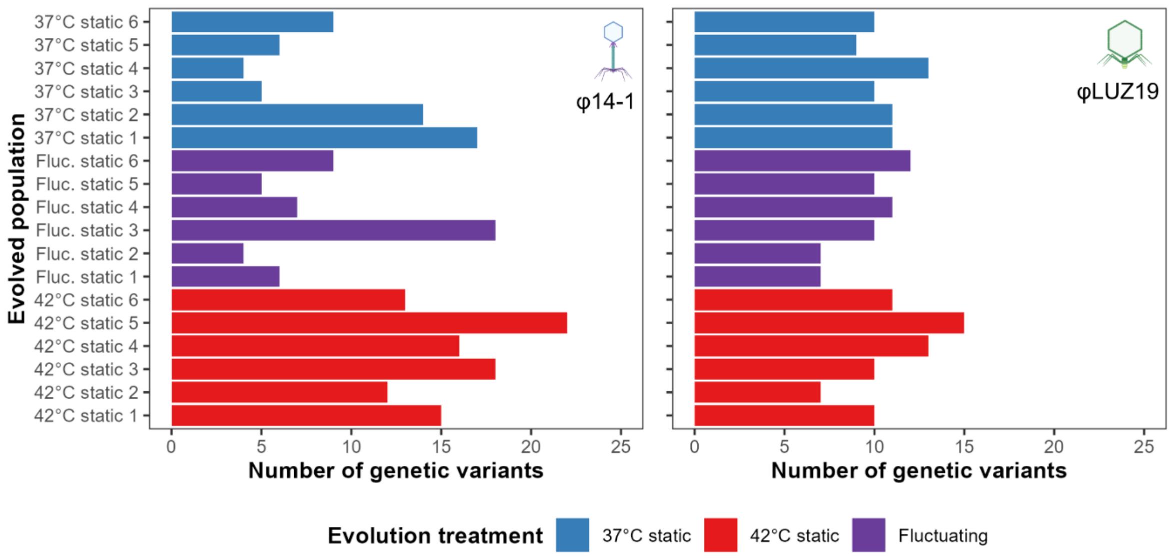

Figure S2. Number of genetic variants across evolved phage populations. Bars show the number of genetic variants for each phage population with φ14-1 shown on the left and φLUZ19 shown on the right. Bars are coloured by evolution treatment. Static temperature data was adapted from ref. (Greenrod et al., 2025).

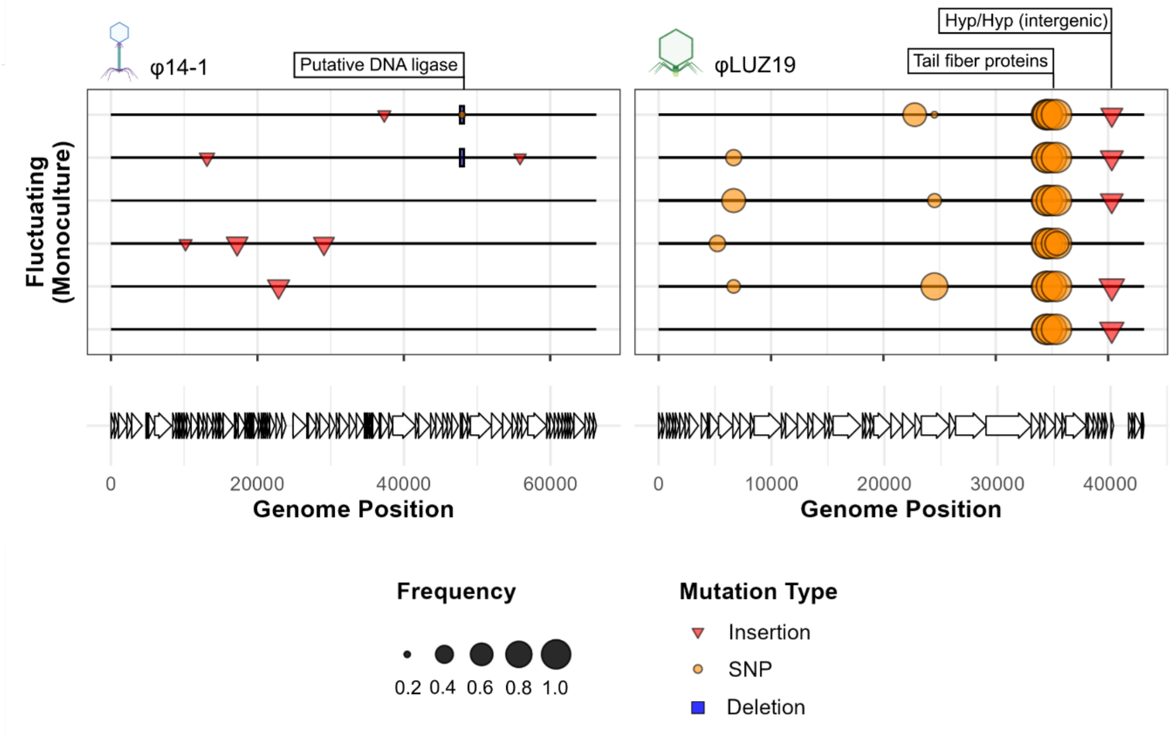

Figure S3. Fluctuating temperatures select for specialist mutations. Mutation plots show genetic variants associated with fluctuating temperatures in monoculture in phage populations. Lines represent individual biological replicates. Symbols within plots show variants across the phage genome at > 20% prevalence and which were not observed in the ancestral population. Length of deletion bars represent the size of deletion except for the φ14-1 deletion at ~48kb which is a 1bp deletion but given a fixed size for visibility. Labels show gene annotations for mutations found in 37°C and 42°C evolved populations (Greenrod et al., 2025). Putative DNA ligase in φ14-1 was originally annotated a hypothetical protein but has high homology to Pseudomonas phage PhL_UNISO_PA-DSM_ph0031 DNA ligase protein.

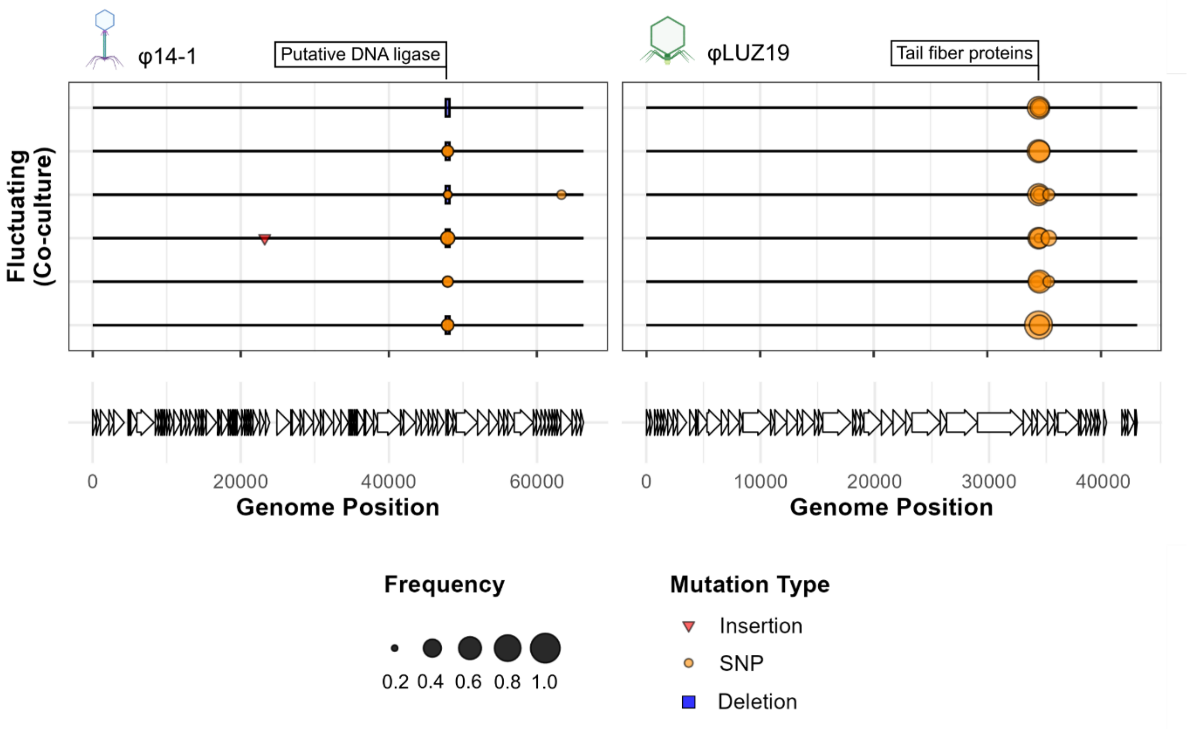

Figure S4. Competition restricts the acquisition of high frequency mutations. Mutation plots show genetic variants associated with fluctuating temperatures in co-culture in phage populations. Lines represent individual biological replicates. Symbols within plots show variants across the phage genome at > 20% prevalence and which were not observed in the ancestral population. Length of deletion bars represent the size of deletion except for the φ14-1 deletion at ~48kb which is a 1bp deletion but given a fixed size for visibility. Labels show gene annotations for mutations found in 37°C and 42°C evolved populations (Greenrod et al., 2025). Putative DNA ligase in φ14-1 was originally annotated a hypothetical protein but has high homology to Pseudomonas phage PhL_UNISO_PA-DSM_ph0031 DNA ligase protein.

Figure legend references

Greenrod, S. T. E., Cazares, D., Ślesak, W., Hector, T. E., MacLean, R. C., & King, K. C. (2025). Evolutionary rescue accelerates competitive exclusion in a parasite community (p. 2025.09.25.678511). *bioRxiv*. https://doi.org/10.1101/2025.09.25.678511
